# LTr1 alleviates DSS-induced ulcerative colitis by modulating macrophages to inhibit M1 polarization and associated inflammatory responses

**DOI:** 10.3389/fimmu.2025.1651922

**Published:** 2025-09-25

**Authors:** Wenjing Zhu, Qian Cheng, Chang Liu, Jiacheng Qian, Jia Xu, Xinyuan Wang

**Affiliations:** ^1^ College of Art, Jiangsu Open University, Nanjing, China; ^2^ Department of Oncology, Nantong University, Nantong, China; ^3^ Hamilton Institute, Maynooth University, Maynooth, Kildare, Ireland; ^4^ Yancheng Key Laboratory of Molecular Epigenetics, Yancheng Medical Research Center, Nanjing University Medical School, The First People’s Hospital of Yancheng, Yancheng, China; ^5^ Department of Human Anatomy, School of Basic Medicine, Nanjing Medical University, Nanjing, China; ^6^ School of Biochemistry and Immunology, Trinity Biomedical Sciences Institute, Trinity College Dublin, Dublin, Ireland

**Keywords:** colitis, LTr1, macrophages, M1 polarization, network pharmacology

## Abstract

**Introduction:**

Ulcerative colitis (UC) is a chronic inflammatory bowel disease marked by recurrent mucosal inflammation, leading to symptoms such as bloody diarrhea and weight loss, which severely impair patients’ quality of life. Current treatments are often limited by long-term efficacy and safety concerns. LTr1, a trimeric compound derived from indole-3-carbinol (I3C), has shown anti-cancer potential, but its role in inflammatory diseases remains unclear. This study aims to investigate the protective effects and underlying mechanisms of LTr1 in a dextran sulfate sodium (DSS)-induced colitis mouse model.

**Methods:**

UC was induced by administering 2.5% DSS in drinking water for 7 days, while LTr1 was orally administered at 100 mg/kg daily starting from day 1. Clinical symptoms, histological changes, and pro-inflammatory cytokine levels in the colon and serum were assessed. Macrophage infiltration and polarization in the colon and spleen were analyzed by flow cytometry and qPCR. *In vitro*, the direct effects of LTr1 on macrophage polarization were examined using CCK-8, flow cytometry, and qPCR. Network pharmacology was employed to explore potential molecular mechanisms.

**Results:**

LTr1 significantly alleviated clinical symptoms, reduced histological damage, preserved intestinal barrier integrity, and suppressed the production of inflammatory cytokines. It also inhibited DSS-induced macrophage infiltration and M1 polarization *in vivo*. Moreover, LTr1 directly and effectively suppressed LPS-induced M1 macrophage polarization *in vitro*. Finally, network pharmacology analysis identified TP53, AKT1, HSP90AA1, EGFR, and SRC as potential targets of LTr1 in the context of UC.

**Conclusion:**

These findings indicate that LTr1 exerts protective effects against DSS-induced colitis, at least in part by inhibiting macrophage infiltration and M1 polarization, thereby reducing pro-inflammatory cytokines production. This study provides a theoretical foundation for optimizing UC treatment strategies and highlights LTr1 as a promising candidate for the development of novel UC therapies.

## 1 Introduction

Ulcerative colitis (UC) is a chronic inflammatory bowel disease (IBD) characterized by persistent inflammation of the colonic and rectal mucosa (1). Its long duration and high recurrence rate severely impair patients’ quality of life. The pathophysiology of UC involves a complex interplay of genetic susceptibility, environmental triggers, and immune dysregulation, which compromise the colonic mucosal barrier. This breach permits luminal antigens to penetrate and activate submucosal immune cells, perpetuating a cycle of neutrophil infiltration, epithelial damage, and crypt abscess formation (2, 3). Current therapeutic strategies for UC include 5-aminosalicylic acid medications, glucocorticoids, immunosuppressants, and biologics (4–6). However, these treatments face challenges regarding long-term efficacy and safety, highlighting the need to explore alternative therapeutic options, including natural bioactive compounds (7, 8).

Macrophages play a central role in innate immunity, inflammatory regulation, and tissue repair (9). Their functional diversity is governed by polarization, a dynamic process by which macrophages adopt distinct phenotypes in response to microenvironmental stimuli, including cytokines and microbial products. Broadly, macrophages are classified into two phenotypes: classically activated (M1) and alternatively activated (M2) (10). M1 macrophages, induced by stimuli such as lipopolysaccharide (LPS) (11) and interferon-gamma (IFN-γ) (12), drive a pro-inflammatory response via heightened production of interleukin-1β (IL-1β), IL-6, tumor necrosis factor-α (TNF-α), and inducible nitric oxide synthase (iNOS) (13). Conversely, alternatively activated M2 macrophages, stimulated by IL-4, IL-10, IL-13, or transforming growth factor-β (TGF-β), exhibit anti-inflammatory and tissue-reparative functions. These cells secrete IL-10, express Arginase-1 (Arg-1) and CD206, promoting resolution of inflammation and tissue repair (9). While our present work refers to the M1/M2 classification for descriptive purposes, it is important to acknowledge that macrophage polarization is a highly dynamic and context-dependent process that extends beyond this simplified model.

In the intestinal mucosa, macrophages help maintain immune balance by distinguishing between harmless antigens and harmful pathogens (14). However, in UC, this balance is disrupted: there is a significant increase in the abundance and activation of macrophages and often polarizing macrophages toward the M1 phenotype (15, 16). Studies have shown that macrophages from UC patients exhibit abnormal signaling and functional profiles, resulting in the excessive activation of inflammatory responses and the persistence of tissue damage (17). This includes the production of reactive oxygen species (ROS) and recruitment of other immune cells such as neutrophils, which release ROS and proteases, as well as T lymphocytes to the inflamed mucosa. These processes further propagate inflammation, contributing to the characteristic features of UC, including mucosal ulceration, crypt abscesses, and inflammatory infiltrates. Consequently, macrophages are considered to play a pivotal role in UC and have emerged as a novel target to develop new treatment approaches in IBD (15, 18).

2-(Indol-3-ylmethyl)-3,3′-diindolylmethane (LTr1) is a trimeric compound derived from indole-3-carbinol (I3C), a natural bioactive compound abundant in cruciferous vegetables and well recognized for its anti-cancer properties (19). Numerous studies and meta-analyses have demonstrated an inverse correlation between cruciferous vegetable intake and the risk of various common cancers, such as colorectal (20, 21) and breast cancer (22). Additionally, research has shown that LTr1 can effectively act against acute myelocytic leukemia (AML) cells harboring FMS-like tyrosine kinase 3 (FLT3) receptor mutations by inhibiting FLT3 phosphorylation and the expression of downstream proteins (23). Further investigation revealed that LTr1 exhibits broad-spectrum anti-cancer activity across several cancer cell lines, including MCF-7 (breast cancer), A549 (lung cancer), and HepG2 (liver cancer) (24). Thus, LTr1 is regarded as a potent cancer inhibitor, superior to I3C and 3,3′-diindolylmethane (DIM), the latter being the most active and effective metabolite of I3C, currently undergoing phase II/III clinical trials to assess its efficacy in patients with breast cancer (25, 26). Despite the growing evidence of LTr1’s anti-cancer potential, its therapeutic value in inflammatory diseases such as UC remains unexplored.

To address the pressing need for novel UC therapeutics with improved safety and efficacy, we aimed to explore the potential of LTr1 as a candidate agent in the treatment of UC. Given the central role of macrophages in the pathogenesis of UC and the growing interest in modulating macrophage polarization as a therapeutic strategy, we focused on evaluating the effect of LTr1 on macrophage dynamics in the context of intestinal inflammation. Specifically, we sought to investigate whether LTr1 could alleviate colitis symptoms and modulate macrophage polarization in a DSS-induced murine colitis model. This study provides a foundation for assessing the immunomodulatory potential of LTr1 beyond its known anti-tumor activity and may offer new insights into natural compound-based interventions for inflammatory bowel diseases.

## 2 Materials and methods

### 2.1 Mice

Male BALB/c mice (6 weeks old) were obtained from the Model Animal Genetics Research Center of Nanjing University (Nanjing, China). Mice were housed under standard laboratory conditions with free access to standard pellet chow and sterile, distilled water on a 12/12h light/dark cycle.

### 2.2 DSS-induced colitis model conduction

#### 2.2.1 DSS treatment and LTr1 administration

Mice were allowed to adapt for 7 days with unrestricted access to food and water before the experiments. They were randomly assigned to three groups (n = 5 per group): Control group, DSS group, and DSS + LTr1 group. For induction of colitis according to the references (27, 28), mice were administered with 2.5% (w/v) dextran sulfate sodium (DSS) solutions (Sigma-Aldrich, Cat# 42867) ad libitum for 7 days, followed by normal water for 3 days until being sacrificed under anesthesia. The DSS solution were replaced every two days. For LTr1 treatment, LTr1 (LTr1 was synthesized according to the reference (19), dissolved in corn oil to prepare a stock solution) was administered to mice by oral gavage at 100 mg/kg once daily. Mice in the control and DSS groups received an equal volume of corn oil as vehicle.

#### 2.2.2 Evaluation of disease activity index

During DSS treatment, the severity of UC was assessed using the DAI score system (29), which combines scores for body weight loss, stool consistency, and hematochezia. The DAI scoring criteria are as follows: Body weight loss: 0 for within 1% weight loss; 1 for 1–5% weight loss; 2 for 5–10% weight loss; 3 for 10–15% weight loss; 4 for weight loss >15%. Stool consistency: 0 for normal stool; 2 for soft stool; 4 for watery diarrhea. Hematochezia: 0 for normal stool; 2 for moderate bleeding; 4 for gross bleeding. The average of three scores was expressed as the DAI.

#### 2.2.3 Sample collection

At the end of experiment, mice were sacrificed under isoflurane anesthesia. Mice were placed in an induction chamber with 3% isoflurane in oxygen until complete loss of consciousness and cessation of respiratory movement were observed. Cervical dislocation was then performed as a secondary method to ensure death before tissue collection. The entire colon was collected, and the lengths and weights were measured and photographed. The spleen was also harvested, and the wet weights were recorded.

### 2.3 Histopathological analysis

The colon tissues were fixed in 4% formaldehyde (Sigma-Aldrich, Cat# 1.00496), embedded in paraffin (Sigma-Aldrich, Cat# 327212), and sectioned at 5 μm thickness. Sections were stained with Hematoxylin (Sigma-Aldrich, Cat# H3136) and Eosin (Sigma-Aldrich, Cat# HT110116) (H&E) solution, and then the sections were examined under light microscopy for evaluating the histopathologic changes.

### 2.4 Immunohistochemistry

For immunohistochemical staining, the paraffin-embedded colon tissue sections were deparaffinized, rehydrated, and treated with 3% H_2_O_2_ (Sigma-Aldrich, Cat# H1009) to block endogenous peroxidase activity. Antigen retrieval was performed by microwave heating, followed by blocking with 5% bovine serum albumin (Sigma-Aldrich, Cat# 05470) for 30 minutes. After blocking, sections were incubated overnight with primary antibodies against Clandin4 (dilution 1: 200) (Cell signaling, Cat# 94478), Occludin (dilution 1: 200) (Cell signaling, Cat# 91131), ZO-1 (dilution 1: 300) (Cell signaling, Cat# 13663), CD3 (dilution 1: 100) (Cell signaling, Cat# 78588), and F4/80 (dilution 1:100) (Cell signaling, Cat# 70076) at 4°C. Further, after washing with PBS (Gibco, Cat #14190144), the sections were incubated with polyperoxidase-anti-rabbit IgG secondary antibody (dilution 1:1000) (Sigma-Aldrich, Cat# A8275) at room temperature for 1 h. Nuclei were counterstained with DAPI (4′,6-diamidino-2-phenylindole) (dilution 1:10000) (Thermo Fisher, Cat# D1306), and sections were imaged under fluorescence microscopy.

### 2.5 Periodic acid Schiff and alcian blue staining

Fresh colon was fixed in 10% buffered formalin, embedded in paraffin, sectioned at 5 μm thickness, and stained with Periodic acid-Schiff and Alcian blue stain kit (Abcam, Cat# ab245876) according to manufacturer’s instructions. Finally, all samples were observed and photographed with microscopy and the number of goblet cells was normalized to the number of crypt units.

### 2.6 Determination of cytokines

Cytokines levels were assessed using enzyme-linked immune sorbent assay (ELISA). Serum samples were collected via the orbital venous plexus under isoflurane anesthesia. Additionally, RAW264.7 cells were pre-treated with the indicated concentration of LTr1 for 1h and then stimulated with 1 µg/ml LPS (Beyotime, Cat# S1732) for 24h. Conditioned medium was collected for the further analysis by ELISA. This study employed Tumor necrosis factor-α (TNF-α) (R&D, Cat# MTA00B), Interleukin-6 (IL-6) (R&D, Cat# M6000B), Interleukin-1β (IL-1β) (R&D, Cat# MLB00C), Interleukin-12 (R&D, Cat# M1270) kit to measure the levels of cytokines in colon tissues and cell culture medium.

### 2.7 Quantitative reverse transcription-PCR

Total RNA of colon tissue and treated cells was isolated and purified using the Total RNA Isolation Kit (QIAGEN, Cat# 74104). Reverse transcription was performed using the Prime Script TM RT-PCR kit (Takara, Cat# RR014A), and qRT-PCR was performed using the Biorad CFX Connect (Biorad) following the manufacturer’s instructions. The relative expressions of each target gene mRNA were normalized to the housekeeping gene Hypoxanthine-guanine phosphoribosyltransferase (Hprt) by using the 2^-ΔΔCT^ method (30). The primers for the following genes were synthesized by GenScript Biotech: Mouse *IL-1β*, forward, ATGCCACCTTTTGACAGTGATG, and reverse, TGATGTGCTGCTGCGAGATT; Mouse *IL-6*, forward, TAGTCCTTCCTACCCCAATTTCC, and reverse, TTGGTCCTTAGCCACTCCTTC; Mouse *TNFα*, forward, CCTGTAGCCCACGTCGTAG, and reverse, GGGAGTAGACAAGGTACAACCC; Mouse *IL-10*, TTCTTTCAAACAAAGGACCAGC, and reverse, GCAACCCAAGTAACCCTTAAAG; Mouse *IL-12*, ACGAGAGTTGCCTGGCTACTAG, and reverse, CCTCATAGATGCTACCAAGGCAC; Mouse *IFN-γ*, CAGCAACAGCAAGGCGAAAAAGG, and reverse, TTTCCGCTTCCTGAGGCTGGAT; *Hprt*, forward, GTCCCAGCGTCGTGATTAGC, and reverse, TGGCCTCCCATCTCCTTCA.

### 2.8 Lamina propria lymphocytes preparation

Freshly harvested colon tissue was washed with PBS and cut into 1 x 1cm segments. Epithelial cells were removed by sequential incubation: twice in PBS containing 3 mM EDTA (Sigma-Aldrich, Cat# EDS) for 10 minutes at 37°C, followed by two rounds in RPMI 1640 medium (Thermo Fisher, Cat# 11875093) supplemented with 1% fetal bovine serum (FBS) (Gibco, Cat# 16000044), 1 mM EDTA, and 1.5 mM MgCl_2_ (Sigma-Aldrich, Cat# 208337) for 15 minutes. The remaining tissues were digested in RPMI 1640 medium containing 20% FBS, 100 U/mL collagenase D (Roche, Cat# 11088858001), and 5 U/mL DNase I (Sigma-Aldrich, Cat# D8515) for 90 minutes at 37°C with occasional mechanical disruption via syringe aspiration (40–50 times). The resulting cell suspension was subjected to density gradient centrifugation using a 45%/66.6% discontinuous Percoll gradient (Solarbio) at 2500 rpm for 20 minutes. Viable LPLs were counted using trypan blue exclusion assay.

### 2.9 Splenocyte preparation

Spleens were harvested and mechanically disrupted using the back end of a syringe. The tissue was incubated with 100 U/mL collagenase D and 5 U/mL DNase I in 50 mL of fresh RPMI 1640 medium at 37°C with 5% CO_2_ for 20 minutes. Following digestion, the suspension was filtered through 70–100 μm sterile filters and centrifuged. Red blood cells were lysed using RBC lysis buffer (Biolegend, Cat# 420301) for 5 minutes at room temperature. Cells were then washed with fresh medium, centrifuged at 1500 rpm for 5 minutes, and resuspended in fresh medium. Cell viability was assessed by trypan blue exclusion assay.

### 2.10 Flow cytometry analysis

Isolated LPLs and splenocytes were pre-incubated with monoclonal antibody 2.4G2 (anti-mouse CD16/CD32 mAb) (BD Biosciences, Cat# 553141) to block Fcγ receptors. Cells were then stained with fluorochrome-conjugated monoclonal antibodies in PBS containing 2 mM EDTA and 2% FBS for 40 minutes. Cells were analyzed on an LSRFortessa II instrument (BD Biosciences) with FlowJo software (TreeStar). The following antibodies were used for flow cytometry: CD3-Brilliant Violet 510 (eBiosciences, Cat# 464882), CD11b-Brilliant Violet 421 (Biolegend, Cat# 101235), CD19-Brilliant Violet 650 (BD Biosciences, Cat# 563235), CD45-FITC (Biolegend, Cat# 103108), F4/80-PE (BD Biosciences, Cat# 565410). iNOS-APC (Miltenyibiotec, Cat# 130-116-423), CD80-Brilliant Violet 711 (Biolegend, Cat# 104743), CD206-PE/Cyanine7 (Biolegend, Cat# 141720).

### 2.11 Cell culture

RAW264.7 murine macrophage cells were obtained from ATCC and cultured in high-glucose Dulbecco’s modified Eagle’s medium (DMEM) (Sigma-Aldrich, Cat# D5796) with 10% (v/v) FBS, 100 U/ml Penicillin and 100 U/ml Streptomycin (Gibco, Cat# 12090216). Cells were cultured in a 37°C humidified chamber under a 5% CO_2_ atmosphere.

### 2.12 Screening of cellular drug delivery concentrations

RAW264.7 cells were seeded at a density of 5000 cells per well in 96-well plates. Following 24h of incubation, cells were treated with indicated concentrations of LTr1 for 24h. CCK-8 solution (MedChemExpress, Cat# HY-K0301) was added to the wells and the plates were returned to the 37°C humidified chamber under a 5% CO_2_ atmosphere for 1h. The absorbance (A) values of cells were assessed at 450 nm using microplate reader, and the cell viability was computed. Cell viability (%) = (A sample − A blank)/(A control − A blank) * 100%. The half maximal inhibitory concentration (IC50) of LTr1 on cell viability was determined by interpolation from dose-response curves.

### 2.13 Network pharmacology analysis of LTr1 to colitis

#### 2.13.1 Collection of UC-related therapeutic target genes

Therapeutic target genes of UC were collected from Genecards (https://www.genecards.org/) and DisGeNET (https://www.disgenet.org/) databases using the keywords “Ulcerative Colitis”. Upon integration of the data and the removal of duplicated target genes, a comprehensive set of target genes associated with UC was established.

#### 2.13.2 Target prediction of LTr1

The SMILES chemical structure of LTr1 was retrieved from the PubChem database (https://pubchem.ncbi.nlm.nih.gov/). This structure was utilized as input in the chEMBL (https://www.ebi.ac.uk/chembl/), and DGIdb (https://www.dgidb.org/) databases to predict target genes. Upon integration of the data and the removal of duplicated target genes, a comprehensive set of LTr1-associated target genes was established.

#### 2.13.3 Prediction of target genes of LTr1 to UC

The integration of 302 LTr1-associated target genes and 5752 UC-related genes, a subset of 189 genes were found to be shared among these datasets and delineated with the Venn diagram plotted by the R language Venn Diagram package for intuitive vision.

#### 2.13.4 Conducting protein-protein interaction network

The identified 189 intersecting target genes were analyzed for PPI network by using the STRING database (https://cn.string-db.org/). The PPI network was visualized through graphical representation using Cyto-scape software (version 3.9.1). Concurrently, the Cytohubba plugin was employed, utilizing the Maximum Clique Centrality (MCC) algorithm to identify the top 20 core target proteins. The Layout section was configured with the Degree Sorted Circle layout.

#### 2.13.5 Function and pathway enrichment analysis

The R software (version 4.2.0) was utilized to install “colorspace,” “stringi,” and “ggplot2” packages. The Bioconductor package, encompassing “DOSE,” “clusterProfiler,” and “enrichplot,” was applied to perform Gene Ontology (GO) and Kyoto Encyclopedia of Genes and Genomes (KEGG) enrichment analyses on the identified Top 20 intersecting target genes. The “enrichGO” function was employed for Gene Ontology (GO) enrichment analysis, with parameters set as OrgDb = “org.Hs.eg.db,” keyType = “ENTREZID,” and ont = “ALL.” Additionally, the “enrichKEGG” function was utilized for the Kyoto Encyclopedia of Genes and Genomes (KEGG) enrichment analysis, with parameters set as organism = “hsa” and keyType = “kegg.” The filter of the P value for both functions was set to 0.05. The top 10 enrichment results from GO enrichment and KEGG enrichment were visualized as a dot plot and a bar chart, respectively.

### 2.14 Statistical analysis

Data were analyzed using a one-way analysis of variance (ANOVA) and an LSD multiple comparison test to determine statistical differences between groups. All statistical analyses were performed using IBM SPSS Statistics software 25.0, and graphs were prepared using GraphPad Prism software 10.2. All experimental data were expressed as mean ± standard deviation (SD) from at least three independent experiments, with statistical significance set at *p*-value < 0.05.

## 3 Results

### 3.1 LTr1 reversed the symptoms and histological damage in DSS-induced colitis in mice

**Figure 1:**
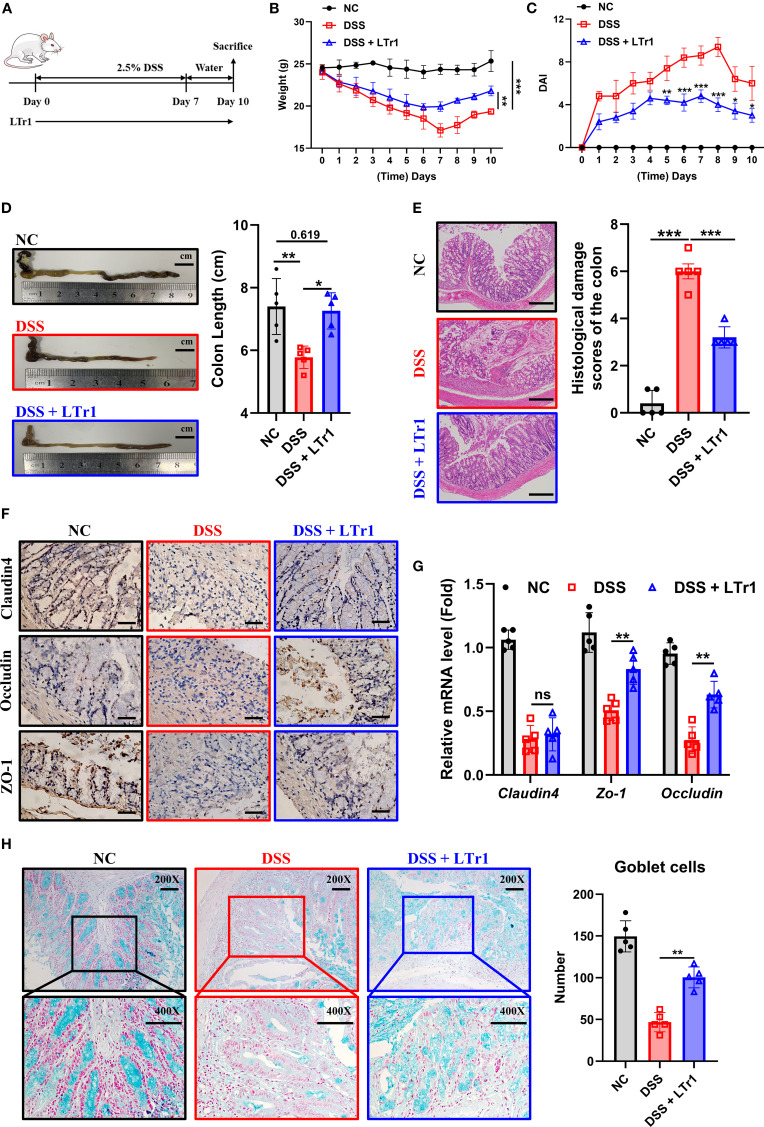
Protective effect of LTr1 in DSS-induced murine colitis. **(A)** Experimental procedure diagram. **(B)** Body weight changes of mice during the experimental period. **(C)** Clinical symptoms of DSS-induced colitis were determined by the disease activity index (DAI). **(D)** Representative images of the colons and colon length analysis of mice. **(E)** H&E staining and corresponding histological damage scores of mice colon tissue. Scale bars represent 100μm. **(F)** Immunohistochemical staining analysis of Claudin-4, Occludin, and ZO-1 protein expression in colon tissue. Scale bars represent 100μm. **(G)** mRNA expression levels of *Claudim4*, *Occludin*, and *Zo-1* in colon tissue determined by quantitative RT-PCR. **(H)** AB-PAS staining (200X and 400X) and goblet cells number counting of mice colon tissue. All experiments were performed with n = 5 mice per group and presented as means ± SD. Statistical analysis was performed using one-way ANOVA followed by LSD multiple comparisons test. *p < 0.05, **p < 0.01, ***p < 0.001.

To investigate the therapeutic potential of LTr1 in UC, we initially established a DSS-induced murine colitis model (**Figure 1A**). Mice receiving 2.5% DSS in drinking water for 7 days exhibited characteristic disease progression, including progressive body weight loss (**Figure 1B**), developed severe colitis, as indicated by an elevated disease activity index (DAI) score (**Figure 1C**), reduced colon length (**Figure 1D**), and extensive histopathological damage (**Figure 1E**) compared to the control group. Notably, LTr1 treatment effectively reversed these colitis-induced effects, with the most pronounced effects observed in body weight and colon length. Specifically, the extent of DSS-induced weight loss was reduced by approximately 50%, and the colon length was restored to a level comparable to that of the healthy control group. After confirming that LTr1 plays a protective role in the DSS-induced colitis model, we further examined the expression of tight junction proteins in intestinal tissues to test the gut barrier function. DSS exposure led to a significant reduction in tight junction proteins Claudin4, Occludin, and ZO-1 expression levels, which was reversed by LTr1 treatment (**Figure 1F**). Similarly, RT-PCR analysis further confirmed that DSS exposure led to a more than 50% decrease in *Claudin-4*, *Occludin*, and *ZO-1* mRNA expression, and LTr1 treatment reversed the transcriptional downregulation of *ZO-1* and *Occludin* mRNA. (**Figure 1G**). In addition, goblet cells play an important role in maintaining intestinal homeostasis and repair, PAS staining revealed more than 60% depletion of goblet cells in DSS-treated mice compared to the control group, which was significantly reversed by LTr1, indicating its role in preserving mucosal integrity (**Figure 1H**). Collectively, these results indicate that LTr1 can effectively protect mice from clinical manifestations of colitis and intestinal tissue damage due to DSS.

### 3.2 LTr1 reduces local and systemic inflammation in DSS-induced colitis

**Figure 2:**
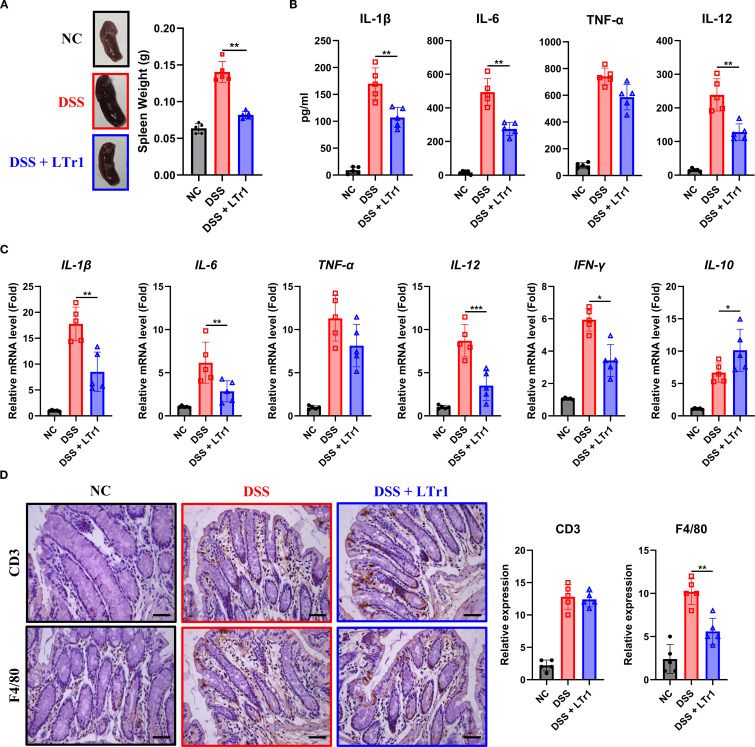
Anti-inflammatory effects of LTr1 in DSS-induced colitis. **(A)** The representative images of the spleen and quantitative analysis of spleen weight. **(B)** ELISA analysis of IL-1β, IL-6, TNFα, and IL-12 in mice ocular blood serum. **(C)** mRNA expression levels of IL-1β, IL-6, TNFα, IL-12, IFN-γ, and IL-10 in colon tissue were determined by quantitative RT-PCR. **(D)** Immunohistochemical staining analysis of CD3, and F4/80 in colon tissue. Scale bars represent 100μm. All experiments were performed with n = 5 mice per group and presented as means ± SD. Statistical analysis was performed using one-way ANOVA followed by LSD multiple comparisons test. *p < 0.05, **p < 0.01, ***p < 0.001.

Dysregulated immune response constitutes a hallmark of UC pathogenesis. Our results demonstrated that DSS-induced colitis was associated with pronounced splenomegaly, as reflected by a spleen weight approximately threefold higher than that of normal controls. Remarkably, LTr1 treatment restored spleen weight to levels comparable to the control group (**Figure 2A**). Given the spleen’s critical role in immune regulation, we hypothesized that LTr1 could modulate the inflammatory response induced by DSS. To assess the anti-inflammatory effect of LTr1, we performed ELISA assay to determine the protein levels of cytokines present in the serum and RT-PCR was conducted to determine the mRNA level of cytokines in the colon of the mice. ELISA results showed serum levels of pro-inflammatory cytokines IL-1β, IL-6, IL-12, and TNF-α were significantly increased in the DSS group compared to those in the control group. LTr1 treatment led to an approximate 50% reduction in the levels of IL-1β, IL-6, and IL-12, although TNF-α levels remained unchanged (**Figure 2B**). Concordantly, RT-PCR results indicated that DSS-induced mRNA transcription of pro-inflammatory cytokines in colon tissue, including IL-1β, IL-6, IL-12, and IFN-γ were inhibited by the LTr1 treatment, while TNF-α expression was unaffected (**Figure 2C**). Additionally, the mRNA expression of the anti-inflammatory cytokine IL-10, whose expression was significantly increased by LTr1 treatment compared to the DSS group (**Figure 2C**). A large infiltration of immune cells in intestinal tissues is another characteristic of colitis. To further confirm the anti-inflammatory effect of LTr1, we used immunohistochemistry to detect the infiltration of immune cells in colon tissues. The results demonstrated that DSS stimulation significantly enhanced the infiltration and accumulation of CD3^+^ T cells and F4/80^+^ macrophages in intestinal tissue compared with the control group. Notably, LTr1 treatment failed to reduce DSS-induced T cells accumulation, but significantly reduced macrophages infiltration (**Figure 2D**) in colon tissues. These findings suggest thatxLTr1 confers a potent anti-inflammatory effect in DSS-induced colitis, potentially by modulating cytokine production and macrophage infiltration.

### 3.3 LTr1 reduces macrophage infiltration and M1 polarization

**Figure 3:**
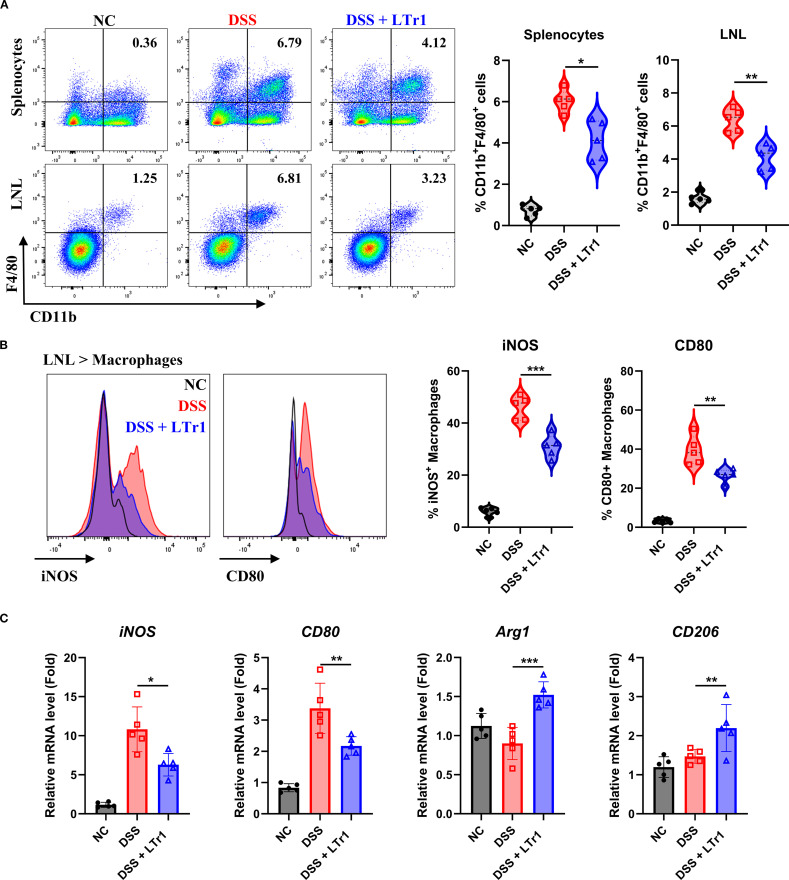
LTr1 reduces DSS-induced macrophage infiltration and M1 Polarization. **(A)** The proportions of macrophages in the splenocytes and LNLs were determined by flow cytometry analysis. **(B)** Protein expression levels of iNOS and CD80 in macrophages of LNLs, along with the percentage of iNOS^+^ or CD80^+^ macrophages as determined by flow cytometry. **(C)** mRNA expression levels of *iNOS*, *CD80*, *Arg1*, *CD206* in colon tissue determined by quantitative RT-PCR. All experiments were performed with n = 5 mice per group and presented as means ± SD. Statistical analysis was performed using one-way ANOVA followed by LSD multiple comparisons test. *p < 0.05, **p < 0.01, ***p < 0.001.

Macrophages play a crucial role in maintaining homeostasis and the development of inflammation in gut (14). Immunohistochemical analysis revealed reduced macrophage infiltration in intestinal tissues following LTr1 treatment (**Figure 2D**). To further explore the impact of LTr1 on macrophage dynamics, we used flow cytometry to quantify macrophage infiltration in spleen and colon tissues. The gating strategy is detailed in **Supplementary Figure 1**, and macrophages were identified as ZombieNIR^-^CD45^+^CD3^-^CD19^-^ CD11b^+^F4/80^+^ cells. Under physiological conditions, macrophages constituted approximately 1% of the cellular population in spleen and colon tissues. DSS challenge elevated the proportion of macrophages to about 7% in both tissues while LTr1 treatment markedly reduced this increase in both spleen and colon tissue (**Figure 3A**). Dysregulation of macrophage polarization plays a critical role for the development of UC (15). To assess this, we examined colonic infiltrating macrophages, using iNOS and CD80 as M1-polarization markers. Flow cytometry revealed that only approximately 5% of macrophages were iNOS^+^, while around 2% were CD80^+^ in the control group, while DSS-induced macrophages exhibited an M1-polarized phenotype, with over 50% iNOS^+^ and 40% CD80^+^ macrophages. In contrast, LTr1 treatment significantly reduced the proportion of both iNOS^+^ and CD80^+^ macrophages, along with a decrease in their protein expression levels (**Figure 3B**). RT-PCR analysis corroborated these findings, the mRNA levels of iNOS and CD80 were reduced, while the mRNA levels of M2-polarization markers CD206 and Arg1 were increased after the LTr1 treatment compared to the DSS group (**Figure 3C**). These data suggest that LTr1 not only inhibits macrophage infiltration but also promotes a shift from pro-inflammatory M1 to anti-inflammatory M2 phenotypes in DSS-induced colitis.

### 3.4 LTr1 directly suppresses M1 polarization in macrophages *in vitro*

**Figure 4:**
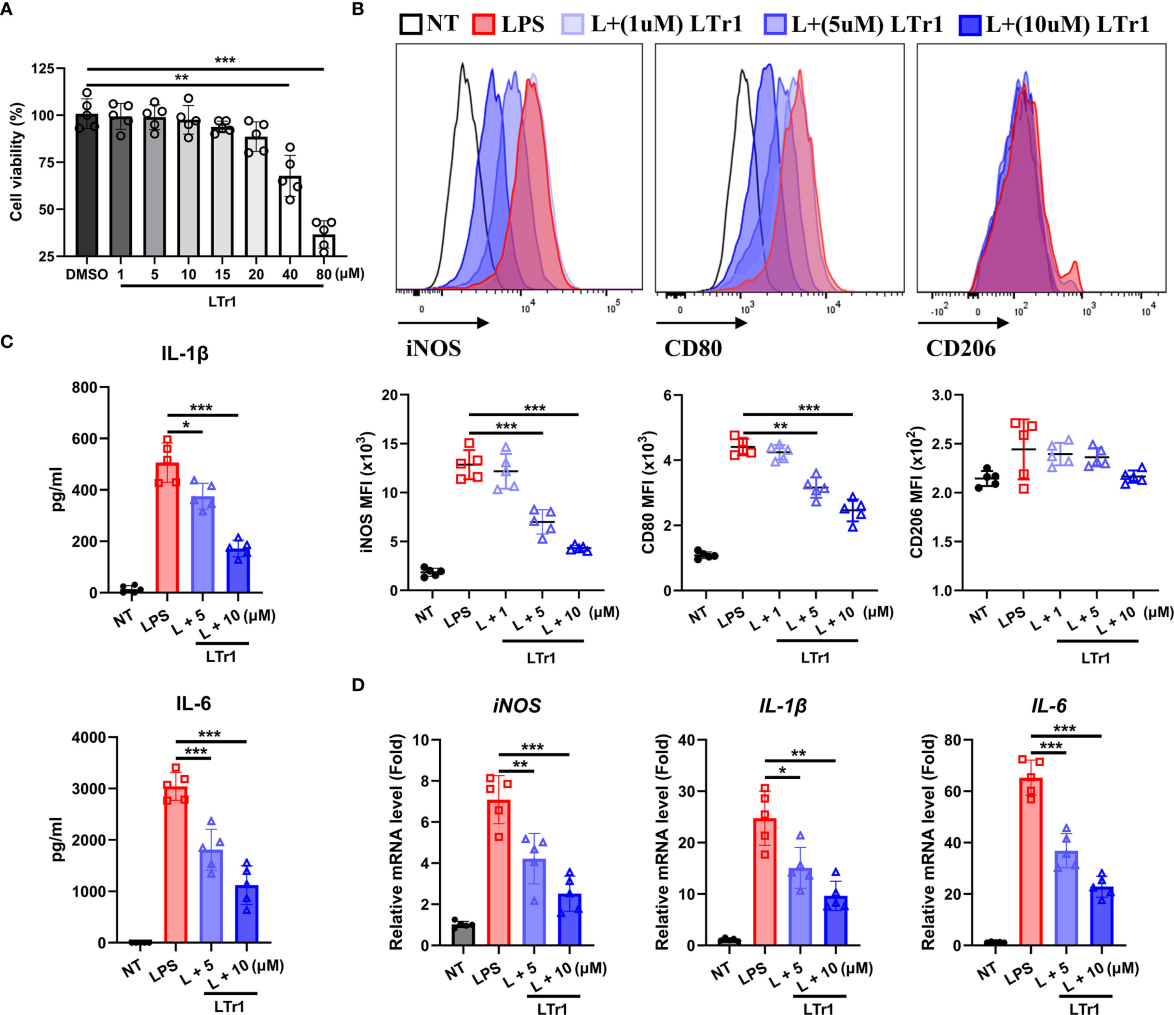
LTr1 directly acted on macrophages. **(A)** Cytotoxicity of LTr1 to RAW264.7 cell line was determined by CCK8 cell viability assay. **(B)** Protein expression levels of iNOS, CD80, and CD206 in RAW264.7 cell line was determined by flow cytometry. **(C)** ELISA analysis of IL-1β and IL-6 in cell culture medium. **(D)** mRNA expression levels of *iNOS*, *IL-1β*, and *IL-6* in colon tissue determined by quantitative RT-PCR. Data were collected from at least three independent experiments and presented as means ± SD. All data statistical differences were evaluated using Permutation test and Bonferroni correction. *p < 0.05, **p < 0.01, ***p < 0.001.

To determine whether LTr1 directly influences macrophage polarization, we conducted an *in vitro* experiment by using the murine macrophage RAW264.7 cell line. Cell viability assays confirmed that LTr1 up to 20 μM had no cytotoxic effect on cell viability (**Figure 4A**). We then established an M1-like inflammation model in RAW264.7 cells by stimulating cells with LPS to mimic the *in vivo* DSS-induced colitis model. Flow cytometry analysis showed that LPS robustly induced iNOS and CD80 expression with minimal effects on CD206, indicative of M1 polarization compared to NT. LTr1 significantly inhibited LPS-induced cellular inflammation by downregulating iNOS and CD80 in a concentration-dependent manner, and notably, treatment with 10 μM LTr1 reduced the expression of iNOS and CD80 by over 50%, while showing no notable effect on CD206 expression (**Figure 4B**). Additionally, LTr1 treatment also inhibited the LPS-induced pro-inflammatory cytokine IL-1β and IL-6 production (**Figure 4C**), as well as suppressed LPS-induced transcriptional activation of pro-inflammatory genes, including iNOS, IL-1β, and IL-6 (**Figure 4D**). These results confirm that LTr1 directly restrains M1 polarization and inflammatory cytokine expression in macrophages under inflammatory conditions.

### 3.5 Network pharmacology analysis of LTr1 in colitis

**Figure 5:**
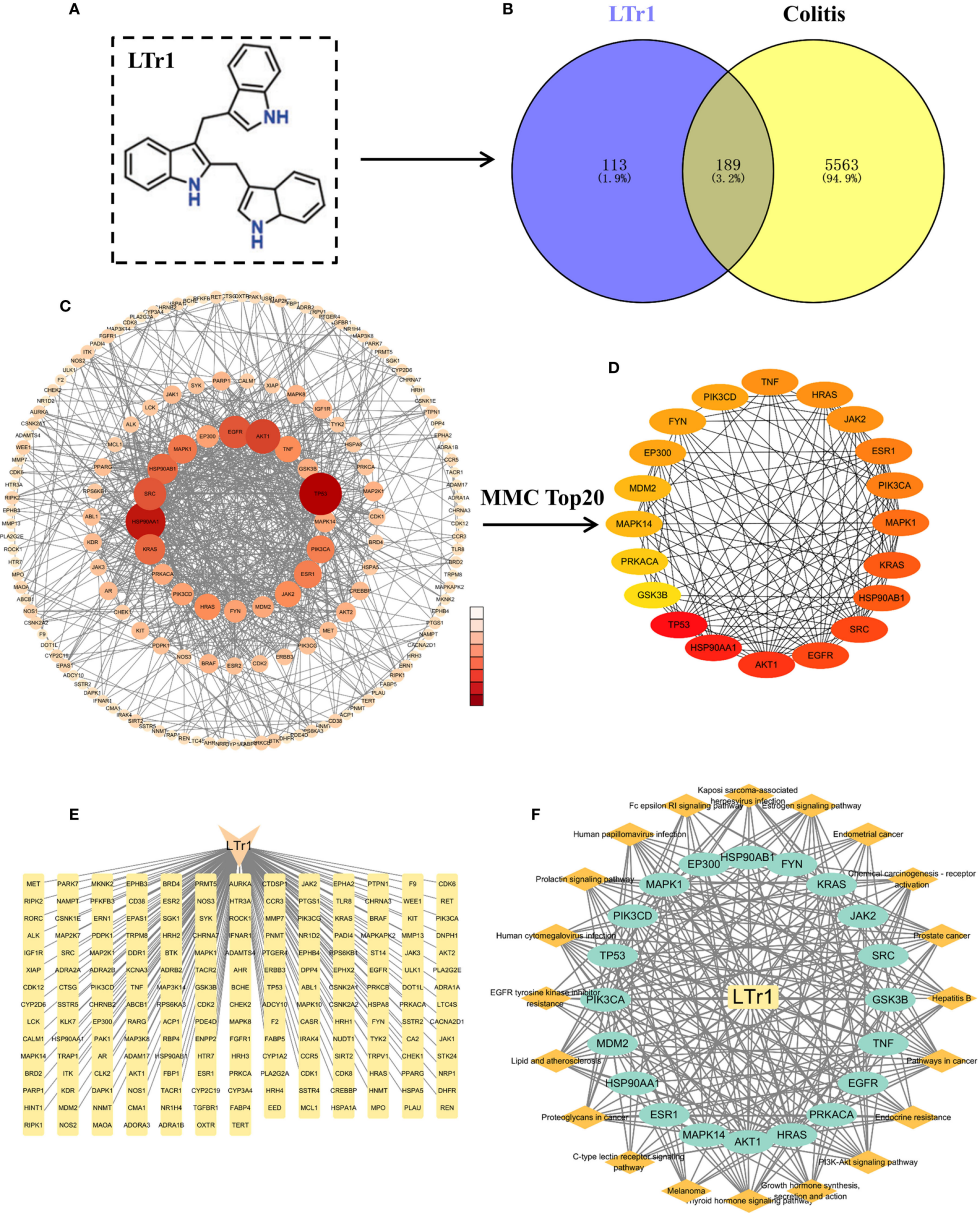
Network pharmacology analysis of LTr1 associated proteins for treating UC. **(A)** The chemical structure of LTr1. **(B)** Venn diagram for overlap between LTr1 target genes and UC-associated genes. **(C)** PPI network analysis of 189 LTr1-UC-related target genes. **(D)** PPI network analysis of Top 20 LTr1-UC-related target genes. **(E)** LTr1-target gene network graph. **(F)** LTr1-core target gene-pathway network graph.

To analyze the potential molecular mechanisms underlying LTr1’s therapeutic effects in UC, the network pharmacology analysis was performed to s identify predicted protein targets correlated with LTr1. Firstly, we obtained the SMILES representation of LTr1 from the PubChem database (**Figure 5A**). Computational predictions for target genes were performed using the SwissTargetPrediction and Pharmaceutical Target Seeker databases. After merging both datasets and removing duplicates, we identified 302 potential target genes. To identify UC-associated genes, we retrieved data from the GeneCards (Relevance score ≥ 1) and DisGeNET databases using the keyword “Ulcerative Colitis”. This search yielded 5,332 genes from GeneCards and 1,682 genes from DisGeNET. After consolidating the datasets and removing duplicates, we compiled a total of 5,752 UC-related genes. Subsequently, intersection analysis was performed using VennDiagram to identify shared genes among the 5752 UC-related genes and the 302 LTr1 target genes. This analysis revealed 189 overlapping genes, which were designated as key targets for further investigation (**Figure 5B**, **Figure 5C**) (**Supplementary Table S1**). Furthermore, we used the CytoHubba plugin with the Maximum Neighborhood Component (MCC) algorithm to identify the top 20 core target genes. In this visualization, nodes transition from orange to red, with darker colors indicating a higher correlation coefficient of action (**Figure 5D**) (**Supplementary Table S2**). Additionally, we constructed a compound-target network graph in Cytoscape, consisting of 190 nodes and 189 edges, where nodes represent targets and edges indicate the interactions between components and targets (**Figure 5E**). Finally, we built a constituent-core target-pathway network to illustrate the relationship between target constituents, core targets and their associated pathways. As shown in **Figure 5F**, the component-core target-pathway interaction network has 41 nodes and 268 edges. The above results suggest that the target ingredient assists in the treatment of ulcerative colitis through a multi-targeted synergistic effect.

### 3.6 Analysis of GO function and KEGG enrichment analysis of 20 core target genes

**Figure 6:**
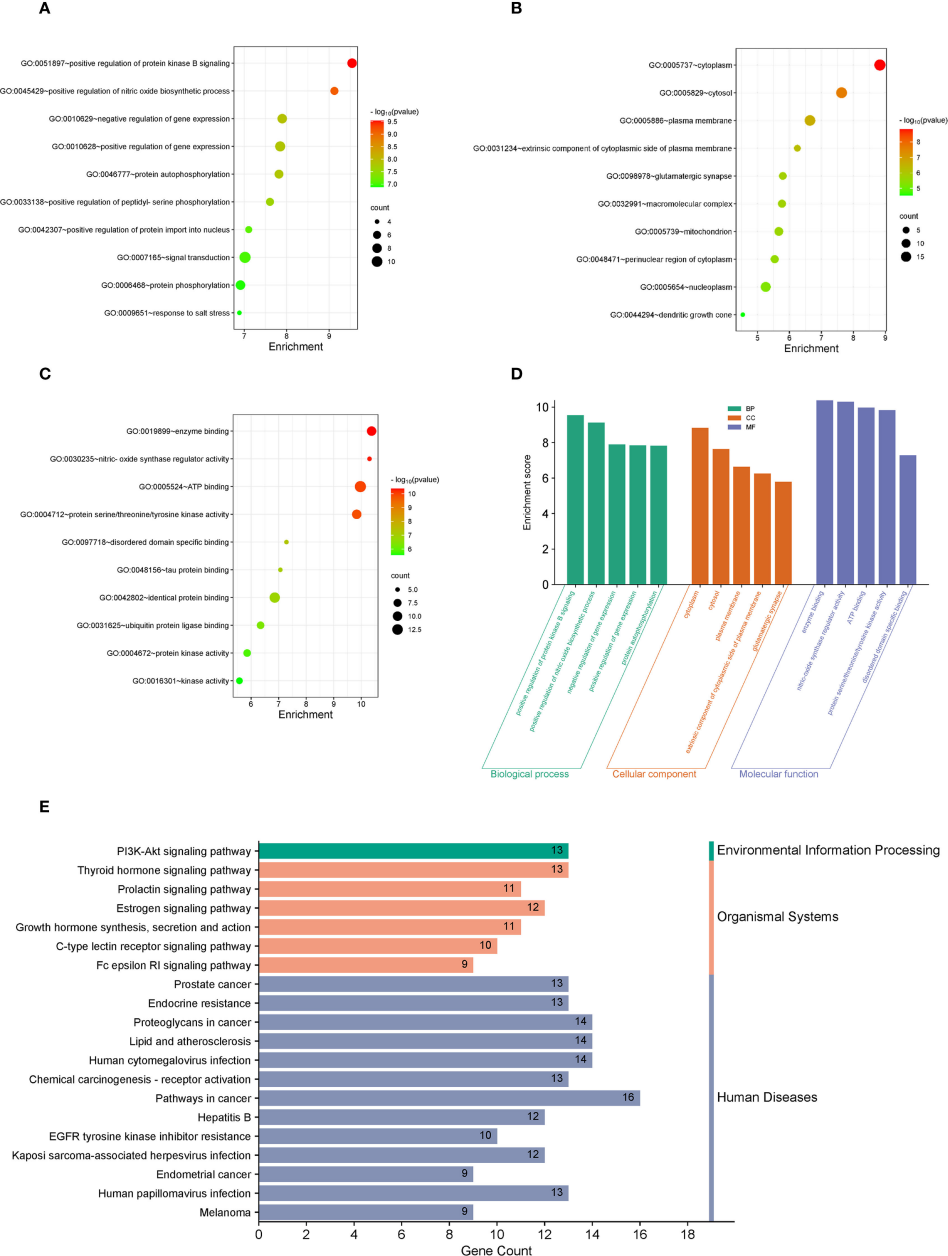
GO and KEGG enrichment analysis of the 23 overlapping target genes. **(A–C)** GO enrichment analysis of LTr1-UC top 20 core target genes. The top 10 enrichment terms of BP show in A, the top 10 enrichment terms of MF show in B, while the top 10 enrichment terms of CC show in C. **(D)** The bar chart histogram map of top 5 pathways enriched in BP, MF, and CC. **(E)** KEGG enrichment analysis of the LTr1-UC top 20 core target genes.

Following the identification of top 20 core target genes associated with both LTr1 and UC, Gene Ontology (GO) and Kyoto Encyclopedia of Genes and Genomes (KEGG) pathway enrichment analysis were performed to investigate the biological roles and related pathways. GO enrichment analysis (*p* < 0.05 and *q* < 0.05) categorized the target genes into three major domains: Biological Process (BP), Molecular Function (MF), and Cellular Component (CC). In total, 214 GO terms were enriched, comprising 245 BP terms, 62 MF terms, and 33 CC terms. Subsequently, we analyzed the data based on *p-value* for the TOP10 in 3 major domains and visualized in a dot plot, the size of the dots indicates the number of genes enriched, while the color of the dots represents the *p-value*. The biological processes mainly involving positive regulation of protein kinase B signaling, positive regulation of nitric oxide biosynthesis process, negative regulation of gene expression, positive regulation of gene expression, protein autophosphorylation, and positive regulation of peptidyl serine phosphorylation (**Figure 6A**). Cellular components mainly involving the cell, cytoplasmic the cellular components mainly involve cell, cytoplasm, plasma membrane, extracellular components of plasma membrane, glutamatergic synapses (**Figure 6B**). The molecular functions mainly involve enzyme binding, nitric oxide synthase regulator activity, ATP binding, protein serine/threonine/tyrosine kinase activity, and disordered structural domain-specific binding (**Figure 6C**). Finally, we brought together the top 5 pathways enriched in the 3 main domains using a bar chart histogram form (**Figure 6D**). For KEGG pathway enrichment analysis (*p-value* < 0.05 and *q* < 0.05), 145 signaling pathways were identified. The top 20 signaling pathways with the lowest *p-value* are illustrated in a chart histogram (**Figure 6E**), and include Endocrine resistance、Thyroid hormone signaling pathway、PI3K-Akt signaling pathway、mTOR signaling pathway、Th17 cell differentiation、MAPK signaling pathway, which was reported to be correlated with inflammation, apoptosis and other physiological activities, and critically impact the pathogenesis of UC.

## 4 Discussion

UC is a chronic inflammatory disorder of the colonic mucosa with an incompletely understood etiology (31). Despite therapeutic advancements including biologics and small molecule inhibitors, approximately 30-40% of patients exhibit suboptimal responses to current therapies (32), and acute enteritis often progresses to chronic enteritis, which in turn develops into colorectal cancer (CAC), there remains an urgent need for the development of novel and effective therapeutic agents with fewer side effects (33). In the present study, we demonstrate for the first time that LTr1 administration significantly alleviates disease severity in the DSS-induced murine colitis model. Specifically, LTr1 treatment notably reduced clinical symptoms such as body weight loss, colon shortening, inflammatory cell infiltration, epithelial injury, and intestinal barrier dysfunction. Notably, mechanistic investigations revealed that LTr1 mediates its therapeutic effects, at least in part, through immunomodulation of macrophage polarization—specifically, by inhibiting M1 pro-inflammatory activity—thus promoting resolution of intestinal inflammation. Finally, a network pharmacology approach was employed to further elucidate the potential molecular mechanisms underlying the protective role of LTr1 in UC. Collectively, our studies indicated that LTr1 may be a potential novel agent for the treatment of UC.

Given the clinical and pathological similarities between DSS-induced colitis in mice and human UC—including symptoms like diarrhea, hematochezia, mucosal inflammation, and barrier disruption—this model serves as a reliable tool for investigating UC pathogenesis and evaluating novel therapeutics (34, 35). LTr1 is a trimeric compound derived from I3C, a naturally occurring anti-cancer molecule found in cruciferous vegetables (24). Previous studies have highlighted its anti-cancer effects in various cancer types and its anti-inflammation effects (19, 23). However, whether LTr1 can treat intestinal diseases (such as UC) caused by inflammation remains undetermined.

In our study, we constructed a DSS-induced colitis model by oral administration of 2.5% DSS in drinking water for 7 days to investigate the protective effect of LTr1 on attenuating UC (28). Our results clearly demonstrated that LTr1 treatment in UC mice showed remarkable therapeutic effects, significantly inhibiting body weight loss, diarrhea, hematochezia, and alleviating colon damage. Moreover, inflammation and intestinal barrier dysfunction are now widely recognized not only as hallmark pathological features of UC, but also as critical therapeutic targets for elucidating its underlying mechanisms and for the development of effective treatment strategies (8, 34). In this context, our findings demonstrated that LTr1 significantly improved histological outcomes by reducing DSS-induced damage to the intestinal villus structure, and partly restored intestinal barrier integrity, as evidenced by increased expression of tight junction proteins such as Occludin and ZO-1, and the preservation of goblet cells in colonic tissues. Interestingly, LTr1 treatment did not effectively restore the DSS-induced downregulation of Claudin-4, suggesting that the regulatory mechanisms governing Claudin-4 expression may differ from those of Occludin and ZO-1, highlighting a potential area for further investigation. Furthermore, the interplay between the intestinal barrier and mucosal immune responses plays a pivotal role in the progression of UC (36). Barrier disruption can lead to dysregulated immune activation and the subsequent overproduction of pro-inflammatory cytokines, such as IL-1β, IL-6, and TNF-α, thereby exacerbating colonic inflammation and tissue injury (37). Notably, LTr1 treatment significantly suppressed the expression of these pro-inflammatory cytokines in both UC mice models. In parallel, LTr1 also promoted the expression of the anti-inflammatory cytokine IL-10, suggesting a dual role in immune modulation and mucosal protection.

Among the immune cells implicated in UC, emerging evidence has identified macrophages as key contributors to the pathogenesis of UC (7, 8, 14). As the largest macrophage population in the body, intestinal macrophages play a central role in regulating mucosal immune responses and promoting tissue repair (38), thereby representing a promising therapeutic target for IBD. Macrophage polarization is closely linked to disease progression in UC (15). Classically activated M1 macrophages are associated with pro-inflammatory responses and have been shown to disrupt epithelial barrier integrity and exacerbate intestinal inflammation (38, 39). In contrast, alternatively activated M2 macrophages exhibit anti-inflammatory properties and contribute to inflammation resolution and mucosal healing (40). The polarization states of macrophages can be characterized by distinct surface markers and cytokine expression profiles: typical M1 markers include iNOS, TNF-α, IL-6, IL-1β, and CD80, whereas M2 macrophages are characterized by the expression of Arg1, IL-10, and CD206 (13, 18). We observed that LTr1 treatment significantly reduced DSS-induced macrophage infiltration in both colon and spleen. In addition, LTr1 effectively inhibited DSS-induced M1 macrophage polarization while promoting M2 polarization, suggesting its immunoregulatory role in modulating macrophage-driven inflammation in UC. To further validate the effect of LTr1 on macrophage polarization, an *in vitro* inflammatory model was established using RAW 264.7 macrophages stimulated with LPS (41). Consistent with previous reports, LPS stimulation induced M1 polarization, characterized by significantly elevated expression of M1 markers such as iNOS and CD80, while M2 marker CD206 levels remained unchanged. Importantly, LTr1 treatment attenuated M1 marker expression and reduced the production of pro-inflammatory cytokines, confirming its regulatory effect on macrophage polarization *in vitro*. Taken together, these findings demonstrate that LTr1 exerts a potent inhibitory effect on M1 macrophage polarization both *in vivo* and *in vitro*, underscoring its therapeutic potential in modulating immune responses during UC.

To further elucidate the mechanisms of anti-inflammatory effect of LTr1 on the UC treatment, potential proteins and signaling pathways were predicted using network pharmacology analyses. Network pharmacology is an interdisciplinary approach integrating high-throughput omics, bioinformatics, and systems biology. It has become instrumental in elucidating the mechanisms of complex biological systems. In recent years, numerous studies have employed this approach to investigate the potential mechanisms by which various natural products exert therapeutic effects in UC (42, 43). This analysis identified that LTr1 targets multiple proteins and signaling pathways relevant to UC, including TP53 (44, 45), AKT1 (46), HSP90AA1, EGFR (47, 48), and SRC (49, 50). Functional enrichment analyses (GO and KEGG) suggested that LTr1 may act through pathways such as PI3K-Akt, mTOR, MAPK, Th17 differentiation, and endocrine resistance—all of which are known to be involved in inflammation, immune regulation, and epithelial regeneration (8, 42, 51). These data support a multi-targeted mechanism of action for LTr1, aligning with the current understanding that UC is driven by complex, interconnected signaling networks rather than single pathways.

However, several limitations should be noted. Although the network pharmacology analysis provided valuable insights, it remains predictive in nature. Therefore, the direct molecular interactions between LTr1 and its proposed targets require further validation through biochemical assays, gene silencing, and target engagement studies. For example, the PI3K-Akt, mTOR, and MAPK signaling pathways have been identified to play critical roles in macrophage M1 polarization (52–54). Thus, elucidating whether LTr1 directly modulates these pathways would significantly enhance our understanding of its pharmacological mechanisms. In addition, as shown in **Figure 3**, LTr1 treatment significantly upregulated the mRNA expression of M2-associated markers Arg1 and CD206, suggesting that LTr1 may not only suppress M1 polarization but also potentially promote M2 polarization—a hypothesis that warrants further investigation. Moreover, in the context of UC, macrophage-mediated pathogenesis involves not only M1 polarization but also the activation of helper T cells (Th cells) by M1 macrophages. This leads to the establishment of a vicious cycle of mutual activation between Th1 cells (secreting IFN-γ) and M1 macrophages, as well as the stimulation of Th17 cells (producing IL-17). These Th17 cells in turn promote the release of matrix metalloproteinases (MMPs) from various cell types, contributing to extracellular matrix degradation and intestinal barrier disruption, thereby perpetuating chronic inflammation and tissue damage (55, 56). Interestingly, our network pharmacology analysis also suggested that LTr1 might modulate Th17 cell differentiation. Therefore, further investigation is required to determine whether LTr1 can directly modulate Th17 cell differentiation, which would provide deeper mechanistic insights into its immunoregulatory functions and therapeutic potential in UC. In addition, Comprehensive pharmacokinetic and safety profiling of LTr1, especially in chronic or relapsing models, is essential before clinical translation. Lastly, confirmation of LTr1’s efficacy and mechanism of action in human-derived systems—such as intestinal organoids or biopsy specimens—will be critical to assess its translational relevance and therapeutic viability in clinical settings.

## 5 Conclusion

**Figure 7:**
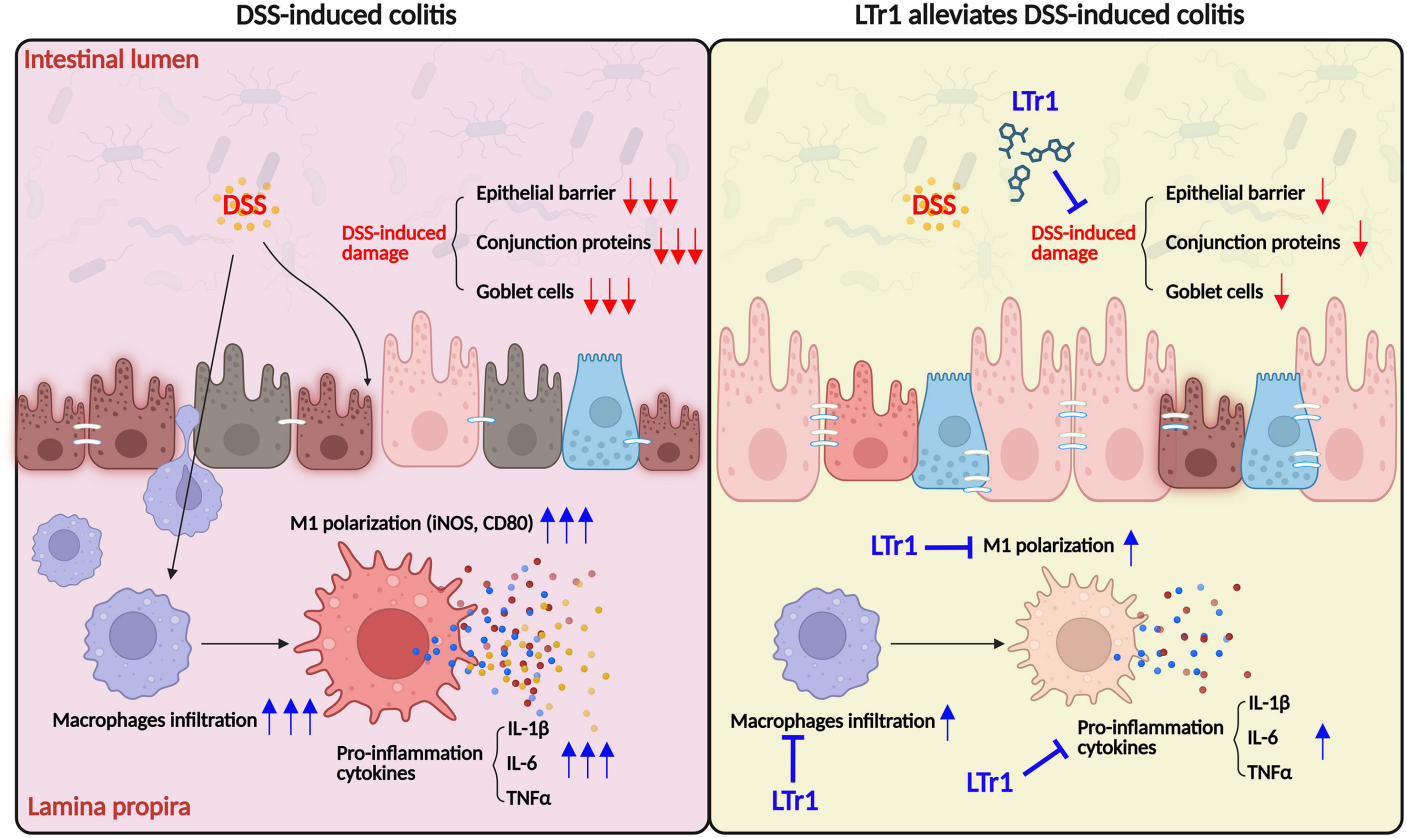
Proposed mechanism of LTr1 in treating DSS-induced colitis by regulating macrophage polarization.

In conclusion, this study demonstrates that LTr1 exerts significant therapeutic effects in a murine colitis model, mainly by attenuating intestinal inflammation and restoring epithelial barrier integrity. Mechanistically, LTr1 modulates the innate immune response by inhibiting macrophage infiltration and suppressing M1 polarization, thereby contributing to the resolution of mucosal inflammation (**Figure 7**). These findings provide new insights into the immunopathogenesis of ulcerative colitis and highlight LTr1 as a promising candidate for developing novel therapeutic strategies that target macrophage-driven inflammation in inflammatory bowel disease.

## Data Availability

The datasets presented in this study can be found in online repositories. The names of the repository/repositories and accession number(s) can be found in the article/**Supplementary Material**.

## Supplementary material

Supplementary Figure 1 Gating strategies for macrophages in spleen and colon. **(A)** Gating strategy for macrophages in spleen (ZombieNIR^-^CD45^+^CD3^-^CD19^-^CD11b^+^F4/80^+^). **(B)** Gating strategy for macrophages in colon tissue (ZombieNIR^-^CD45^+^CD3^-^CD19^-^CD11b^+^F4/80^+^).
