# Supplementary material for: LTr1 alleviates DSS-induced ulcerative colitis by modulating macrophages to inhibit M1 polarization and associated inflammatory responses
Source: Front Immunol. 2025 Sep 25;16:1651922. doi: 10.3389/fimmu.2025.1651922 (PMC12507645; doi:10.3389/fimmu.2025.1651922)
Supplement: Supplementary file 2 [file Table1.docx]

| **Gene Official Symbol** | | | | | | |
| --- | --- | --- | --- | --- | --- | --- |
| CHEK1 | CREBBP | MET | WEE1 | PADI4 | ERBB3 | RPS6KA3 |
| STK24 | HNMT | PARK7 | RET | MAPKAPK2 | DPP4 | CDK2 |
| BRD2 | BRD2 | HSPA5 | MKNK2 | RORC | MMP13 | EPHX2 |
| MAP3K8 | ITK | DHFR | EPHB3 | CSNK1E | DNPH1 | EGFR |
| RBP4 | CLK2 | HINT1 | BRD4 | ERN1 | IGF1R | ULK1 |
| ENPP2 | AKT1 | MDM2 | PRMT5 | EPAS1 | SRC | PLA2G2E |
| FGFR1 | FBP1 | NNMT | AURKA | SGK1 | MAP2K1 | CDK12 |
| FABP5 | ESR1 | CMA1 | CTDSP1 | SYK | DDR1 | CTSG |
| IRAK4 | PRKCA | NR1H4 | JAK2 | ROCK1 | BTK | PIK3CD |
| NUDT1 | PLA2G2A | TGFBR1 | EPHA2 | MMP7 | MAPK1 | TNF |
| TYK2 | CDK1 | FABP4 | PTPN1 | PIK3CG | ADAMTS4 | MAP3K14 |
| CA2 | CDK8 | EED | F9 | KRAS | PTGER4 | GSK3B |
| JAK1 | HRAS | MCL1 | CDK6 | BRAF | EPHB4 | BCHE |
| MAPK14 | PPARG | HSPA1A | RIPK2 | KIT | RPS6KB1 | TP53 |
| TRAP1 | NRP1 | MPO | NAMPT | PIK3CA | ST14 | ABL1 |
| AR | PARP1 | PLAU | PFKFB3 | ALK | JAK3 | CSNK2A1 |
| ADAM17 | KDR | REN | CD38 | MAP2K7 | AKT2 | PRKCB |
| HSP90AB1 | DAPK1 | RIPK1 | ESR2 | PDPK1 | XIAP | DOT1L |
| HTR7 | NOS1 | NOS2 | NOS3 | TRPM8 | ADRA2A | ADRA1A |
| HRH3 | TACR1 | MAOA | HTR3A | HRH2 | ADRA2B | CYP2D6 |
| CYP1A2 | CYP2C19 | ADORA3 | CCR3 | CHRNA7 | KCNA3 | SSTR5 |
| CCR5 | CYP3A4 | ADRA1B | PTGS1 | IFNAR1 | ADRB2 | CHRNB2 |
| SIRT2 | HRH4 | OXTR | TLR8 | PNMT | TACR2 | ABCB1 |
| TRPV1 | SSTR4 | TERT | CHRNA3 | NR1D2 | AHR | HSP90AA1 |
| PAK1 | CHEK2 | ADCY10 | MAPK10 | CSNK2A2 | HSPA8 | PRKACA |
| LTC4S | LCK | KLK7 | EP300 | RARG | ACP1 | PDE4D |
| MAPK8 | F2 | CASR | HRH1 | FYN | SSTR2 | CACNA2D1 |
| CALM1 |  |  |  |  |  |  |

**Table S1 189 overlapping target genes of LTr1 and UC**
