# Supplementary material for: LTr1 alleviates DSS-induced ulcerative colitis by modulating macrophages to inhibit M1 polarization and associated inflammatory responses
Source: Front Immunol. 2025 Sep 25;16:1651922. doi: 10.3389/fimmu.2025.1651922 (PMC12507645; doi:10.3389/fimmu.2025.1651922)
Supplement: Supplementary file 3 [file Table2.docx]

| **Rank** | **geneName** | **Score** |
| --- | --- | --- |
| 1 | TP53 | 62 |
| 2 | HSP90AA1 | 57 |
| 3 | AKT1 | 47 |
| 4 | EGFR | 43 |
| 5 | SRC | 43 |
| 6 | KRAS | 39 |
| 7 | HSP90AB1 | 39 |
| 8 | MAPK1 | 36 |
| 9 | PIK3CA | 34 |
| 10 | ESR1 | 34 |
| 11 | HRAS | 31 |
| 12 | TNF | 31 |
| 13 | JAK2 | 31 |
| 14 | FYN | 24 |
| 15 | EP300 | 24 |
| 16 | PIK3CD | 24 |
| 17 | MDM2 | 22 |
| 18 | MAPK14 | 22 |
| 19 | PRKACA | 21 |
| 20 | GSK3B | 20 |

**Table S2 Top 20 core target genes of LTr1 and UC**
